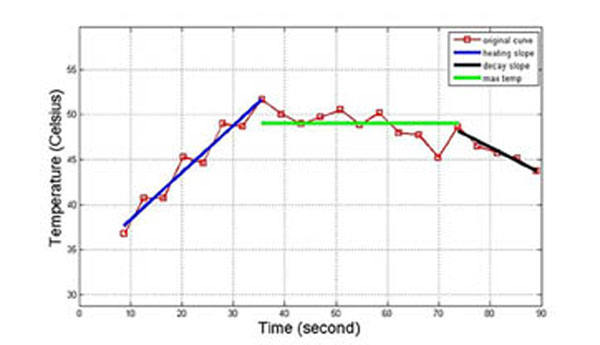# The relationship of area under temperature curve during MR guided HIFU and diffusion coefficient in patient screening

**DOI:** 10.1186/2050-5736-3-S1-P91

**Published:** 2015-06-30

**Authors:** Xuedong Yang, Rong Rong, Jia Liu, Juan Wei, Bilgin Keserci, Xiaoying Wang

**Affiliations:** 1 Peking University First Hospital, Beijing, China; 2 Philips Healthcare, Seoul, Republic of Korea

## Background/introduction

Different type of fibroid shows different response to MR guided HIFU, which is believed to reflect different blood suplly. ADC value derived from low b multiple b value DWI may reflect perfusion information. Our study is to assess the relationship of AUC (area under curve) calculated from the temperature curve during sonication in MR guided HIFU therapy for fibroid and the diffusion coefficient derived from multiple b-values DW-MRI in patient screening.

## Methods

A total of 50 uterine fibroids (diameter: mean, 5.6 cm; range, 3.5–8 cm) in 45 female patients (mean age, 44.8 years) who were given written informed consent underwent MRI screening. In the screening, subjects were positioned prone, feet first, on the 3T MRI scanner (Achieva TX, Philips Medical Systems, Best, the Netherlands) using a 32-channel phased array coil. IVIM MR images were acquired on the axial plan across the uterus. We set different b-values as follows: 0, 25, 50, 75, 100, 150, 200, 500, 800, 1000 (s/mm2). Freehand ROIs circumscribing the fibroids were drawn and data was analyzed by using DWI post- processing software performed in a proprietary programming environment (PRIDE; Philips Medical Systems). For low b-value (<100 s/mm2), monoexp model was used to calculate diffusion coefficient which mainly represented blood perfusion. Thermometry got during the treatments was used to create temperature curves. For better interpretation, two points were chosen on the temperature curve: The first point was the time point at the end of the ascending segment before the temperature got stable. The second point was the time point at the beginning of the descending segment before the temperature began to decay. The Matlab software accompanied with definite integration method was used to analyze the temperature curve to generate area under heating curve (AUC), which was the area under the curve between the beginning point of the curve to the second point. All fibroids were divided into 3 groups according to their AUC: 1) Fibroids with AUC less than 2000°C•s (n=16). 2) Fibroids with AUC between 2000°C•s to 2400°C•s (n=19). 3) Fibroids with AUC more than 2400°C•s (n=15). Independent sample t test was used to test the difference of diffusion coefficient derived from multiple b-values combined with DW-MRI among the 3 group.

## Results and conclusions

Group 1 fibroids with the least AUC (1710.74±189.3°C•s) had the least diffusion coefficient (2.32±0.34μm2/ms). Group 3 fibroids with the most AUC (2798.15±393.7°C•s) had the most diffusion coefficient (2.52±0.19μm2/ms). Group 2 fibroids were with an intermediate AUC and diffusion coefficient (2202.29±135.06°C•s; 2.44±0.22μm2/ms). Fibroids with relatively high AUC which represented more energy during sonication had large diffusion coefficient representative of hyper blood perfusion. However, there was no statistically significant difference of diffusion coefficient among the 3 group (p=0.121). Conclusion: AUC of the temperature curve was related with diffusion coefficient derived from multiple b-values combined with DW-MRI which correlated very well with the biological features of fibroids such as vascularity. Fibroids with high diffusion coefficient representative of hyper blood perfusion need more sonication energy during MR guided HIFU which ensures good therapeutic effects.

**Figure 1:**